# Revitalizing Skin Repair: Unveiling the Healing Power of Livisin, a Natural Peptide Calcium Mimetic

**DOI:** 10.3390/toxins16010021

**Published:** 2023-12-31

**Authors:** Xuehui Zhan, Danni Wang, Hanfei Wang, Hui Chen, Xinyi Wu, Tao Li, Junmei Qi, Tianbao Chen, Di Wu, Yitian Gao

**Affiliations:** 1 Zhejiang Provincial Key Laboratory for Water Environment and Marine, Biological Resources Protection, College of Life and Environmental Science, Wenzhou University, Wenzhou 325035, China; zxh_5513@163.com (X.Z.); danni_wang0113@163.com (D.W.); wanghf@webmail.hzau.edu.cn (H.W.); lt980501@163.com (T.L.); qjm1357@163.com (J.Q.); 2 School of Pharmaceutical Sciences, Wenzhou Medical University, Wenzhou 325015, China; chenhui4649@163.com (H.C.); wuxinyi_wmu@163.com (X.W.); 3 College of Animal Science and Technology, Huazhong Agricultural University, Wuhan 430070, China; 4 Natural Drug Discovery Group, School of Pharmacy, Queen’s University Belfast, Belfast BT7 1NN, UK; t.chen@qub.ac.uk

**Keywords:** trauma repair, peptide, cell migration, angiogenesis, collagen production

## Abstract

When the skin is damaged, accelerating the repair of skin trauma and promoting the recovery of tissue function are crucial considerations in clinical treatment. Previously, we isolated and identified an active peptide (livisin) from the skin secretion of the frog *Odorrana livida*. Livisin exhibited strong protease inhibitory activity, water solubility, and stability, yet its wound-healing properties have not yet been studied. In this study, we assessed the impact of livisin on wound healing and investigated the underlying mechanism contributing to its effect. Our findings revealed livisin effectively stimulated the migration of keratinocytes, with the underlying mechanisms involved the activation of CaSR as a peptide calcium mimetic. This activation resulted in the stimulation of the CaSR/E-cadherin/EGFR/ERK signaling pathways. Moreover, the therapeutic effects of livisin were partially reduced by blocking the CaSR/E-cadherin/EGFR/ERK signaling pathway. The interaction between livisin and CaSR was further investigated by molecular docking. Additionally, studies using a mouse full-thickness wound model demonstrated livisin could accelerate skin wound healing by promoting re-epithelialization and collagen deposition. In conclusion, our study provides experimental evidence supporting the use of livisin in skin wound healing, highlighting its potential as an effective therapeutic option.

## 1. Introduction

The skin, the human body’s largest organ, has the vital functions of protecting the body from external stimulation, sensing temperature changes and pain, regulating humidity, and aiding in the synthesis of vitamin D [1]. Serving as the outer barrier of the body, the skin is vulnerable to damage, and injuries to the skin can disrupt the body’s homeostasis. Therefore, when the skin is damaged, it must be effectively repaired as soon as possible to prevent excessive blood loss and bacterial infection from causing further damage to the body [2]. Skin trauma, which refers to the injury of open wounds, occurs as a result of skin damage. The process of skin trauma healing involves three stages: the inflammatory stage, the proliferative stage, and the remodeling stage. At present, the main clinical treatment for skin trauma primarily includes skin grafting, cell technology, negative pressure wound therapy, electrical stimulation, targeted drugs, growth factors, and other approaches [3]. Despite the development and utilization of a large number of drugs having accelerated the treatment of skin trauma, such as anti-inflammatory drugs and growth factor drugs, there were still some problems, including potential side effects, drug resistance, potential carcinogenic effects, and high costs, etc.

In animals, peptides form an integral part of innate immunity, and they can be short, composed of about 10 amino acids, or much longer, up to 60 amino acids [4]. These named host defensive peptides (HDPs) have a variety of biological activities, and in addition to being antibacterial, anti-inflammatory, and anti-cancer, they can also promote the repair of skin wounds [5]. They achieve this by recruiting inflammatory factors and chemokines, which in turn attract granulocytes and macrophages to the wound site to eliminate invading bacteria and cell debris. For instance, the host defense peptide WHP from the other *odorrana* frog, *Odorrana tormota* can stimulate macrophages to produce chemokines and inflammatory factors, such as CXCL1, CXCL2, CXCL3, TNF-α, IL-1β, and IL-6, through MAPK and NF-κB pathways [6].

During the stage of wound re-epithelialization, keratinocytes proliferate and migrate into the wound to form new epithelium, which later differentiates into the epidermis [7]. For example, the keratinocyte-derived cationic peptide SPINK9 mediates the activation of matrix metalloproteinase (MMP) through purine receptors, and the activation of EGFR further promotes cell migration through ERK [8]. Additionally, during wound healing, fibroblasts secrete collagen and can transform into myofibroblasts to aid in wound contraction. Another example is the peptide AH90, from the frog *Odorrana graham,* which can activate the TGF-β1/Smad signaling pathway in fibroblasts through the MAPK and NF-κB pathways. Once activated, TGF-β1 can upregulate the expression of α-SMA to facilitate the transformation of the fibroblasts into myofibroblasts, ultimately accelerating the wound contraction [9].

HDPs are widely distributed in multicellular organisms, the most prevalent being from the skin of amphibians, such as frogs. Compared with vertebrates, amphibians live in a more complex environment and have more chemically complex skin secretions. The peptides in these skin secretions have different biological activities, including antibacterial and anti-inflammatory activities, among other activities, which are the basis of their significant wound-healing or regeneration ability [10]. In the past few decades, a variety of amphibian skin-derived peptides have been reported. Some of these have been shown to effectively assist the proliferation and migration of human keratinocytes to stimulate the regeneration of epidermis and granulation tissue. The skins of odorous frogs contain extremely rich antimicrobial peptides [11]. For example, RL-QN15, a short peptide isolated from the skin secretion of the frog *Rana limnocharis* has been found to accelerate wound healing by activating the MAPK and Smad signaling pathways [12,13]. Similarly, OA-GL17d, a homodimeric peptide extracted from the skin secretion of the frog *Odorrana andersonii* has been shown to promote skin wound regeneration through the miR-663a/TGF-β1/Smad axis [14]. Additionally, studies have demonstrated the efficacy of fresh frog skin in promoting the wound-healing process [15]. Consequently, amphibian skin secretions are increasingly recognized as valuable sources for the development of regenerative peptides for wound healing. Despite these promising findings, research on the tissue repair aspect of peptides is still in its early stage and needs further exploration.

Recently, peptides containing the classical Bowman–Birk protease inhibitory motif have been identified in the skin secretions of amphibians, mainly from the frog species. We have previously reported the isolation and characterization of a 17-amino acid peptide (livisin) from the skin secretion of *Odorrana livida* [16]. Livisin belongs to the Bowman–Birk trypsin inhibitor family. It is a relatively stable peptide and has antimicrobial activity. In this study, we investigated the wound-healing activity of livisin using both in vitro and in vivo experiments. Futhermore, it was found livisin operated through the activation of the CaSR/E-cadherin/EGFR/ERK signaling pathways, thereby contributing to its wound-healing activity.

## 2. Results

### 2.1. Livisin Promotes Wound Healing In Vitro

#### 2.1.1. Livisin Promotes Cell Migration and Angiogenesis

**Figure 1:**
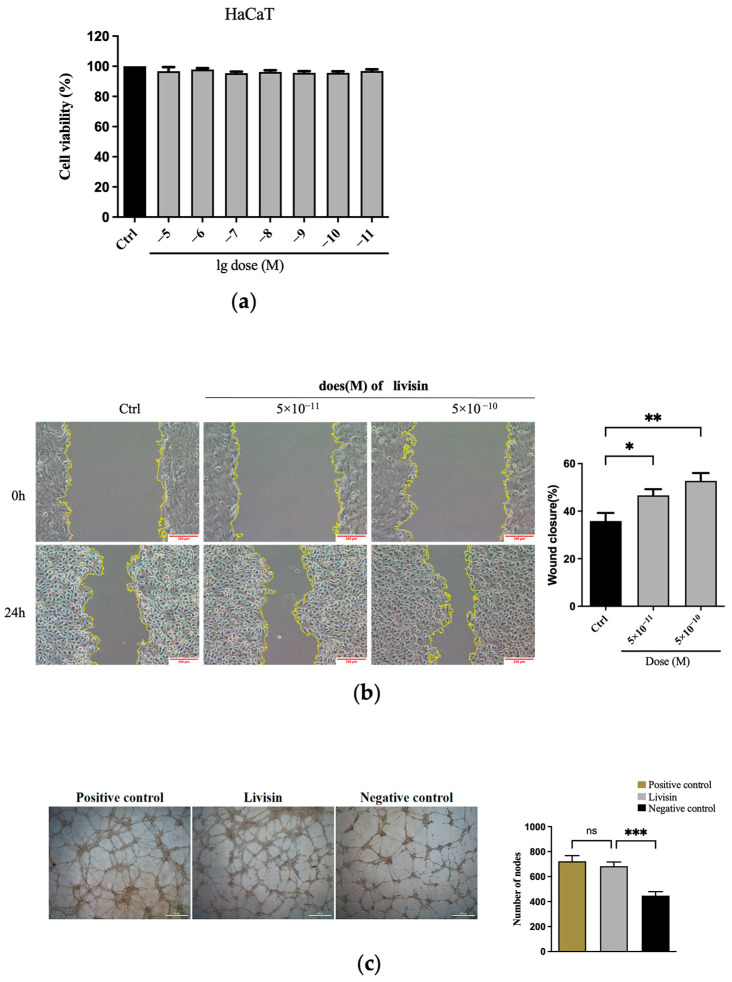
Livisin promotes cell migration and angiogenesis. (**a**) Effect of livisin on keratinocyte proliferation. Keratinocytes were treated with different concentrations of livisin for 24 h, then incubated with MTT for 4 h; (**b**) scratch wound assay of mitomycin C-treated HaCaT cells in the absence and presence of different concentrations of livisin. Images were captured immediately after scratching (0 h) and 24 h. Scale bars: 200 μm. The plot beside the images compares the proportions of wound closure; (**c**) effect of livisin on angiogenesis. HMEC-1 cells were seeded on matrix gels and stimulated with livisin for 18 h. Scale bars: 500 μm. The plot beside the images compares the number of nodes. All graphical data are the means ± SEMs from three determinations. “*”, “**” and “***” indicate significant differences from the control cells at the *p* < 0.05, *p* < 0.01, and *p* < 0.001 levels, respectively. “ns” indicates no significant difference.

The impact of livisin on wound healing was initially evaluated through an MTT assay and a scratch wound assay. The results of the MTT assay showed livisin did not exert any influence on the cell viability of HaCaT, L929, and HMEC-1 cells, suggesting livisin did not promote wound healing by promoting the proliferation of these cells (Figure 1a and Figures S1a and S2a). Additionally, these results also showed livisin had no cytotoxicity in the concentration range of 1 × 10^−5^–1 × 10^−11^ M, which was a good quality in terms of the medicinal properties of livisin. 

In the scratch wound assay, keratinocytes treated by livisin for 24 h displayed rapid coverage of the wound area, with a significantly different effect compared to the untreated group. The migration rates reached 52.74 ± 3.30% at a concentration of 5 × 10^−10^ M, in contrast to 35.87 ± 3.39% in the control group (Figure 1b). Livisin also improved the migration rates of L929 and HMEC-1 cells. Specifically, L929 and HMEC-1 cell migration rates in the livisin treatment group were 28.41 ± 1.66% (Figure S1b) and 43.56 ±1.81% (Figure S2b), respectively, while the control group’s migration rates were 20.54 ± 0.86% (Figure S1b) and 34.23 ± 1.23% (Figure S2b), respectively.

Futhermore, the impact of livisin on angiogenesis, an important phase in wound healing, was studied. The capacity of HMEC-1 cells to form tubes was assessed using the tube formation assay on Matrigel, which served as an in vitro model of angiogenesis. The results indicated a significant increase in the number of lumens and nodes of HMEC-1 cells treated with livisin, which was 1.6 times higher than that of untreated cells. Additionally, the angiogenesis rate was comparable to that of the positive control group (Figure 1c), indicating the efficient stimulation of angiogenesis by livisin.

#### 2.1.2. Livisin Promotes Keratinocyte Migration

**Figure 2:**
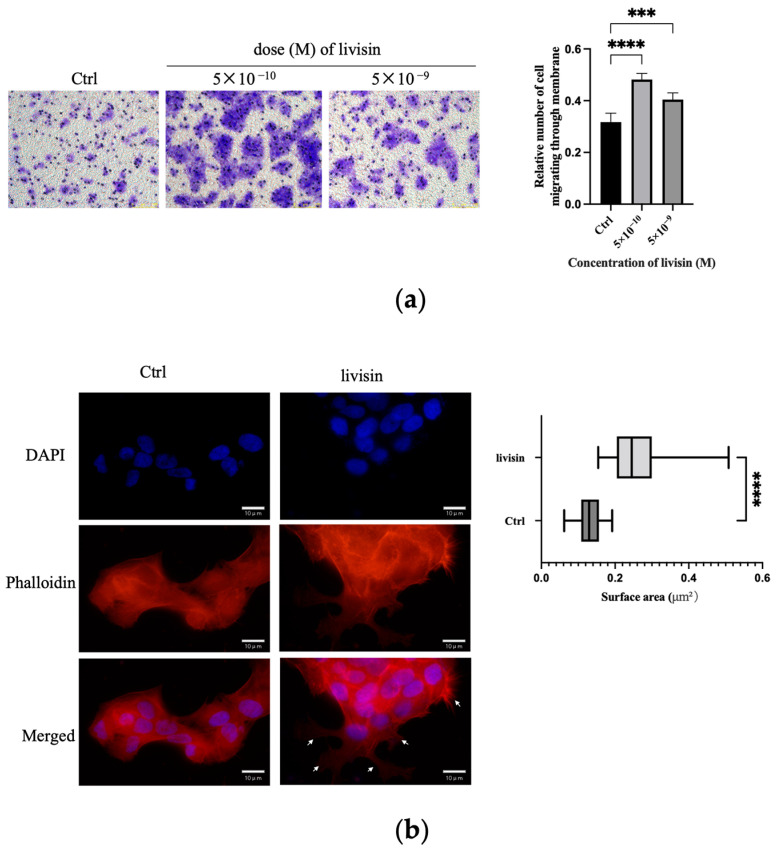
Livisin promotes keratinocyte migration. (**a**) Transwell assay conducted with keratinocytes in the absence and presence of livisin. Scale bar: 100 μm. The plot beside the images compares the proportions of migrated cells. Data in the plot are the mean ± SEM from three independent experiments; (**b**) DAPI staining of HaCaT cells treated without or with 5 × 10^−10^ M livisin for 3 h. Cell nuclei were stained with DAPI whereas the F-actin proteins were stained with TRITC-labeled phalloidin. The cells were observed under a fluorescent microscope. The white arrow marks the flake pseudopods. Scale bars: 10 μm. The plot beside the image shows the area of keratinocytes lamellar or needle-like pseudopodia assessed by phalloidin staining. Data are the mean ± SD of at least three independent experiments. For the cell counting, at least 100 cells were counted for each group. “***” and “****” indicate significantly different from the control at the *p* < 0.001 level and *p* < 0.0001 levels, respectively.

The impact of livisin on cell migration was further assessed using the transwell assay. Upon the addition of various concentrations of livisin (ranging from 5 × 10^−10^ M to 5 × 10^−9^ M) to the lower chambers, a significant increase in the number of keratinocytes crossing the transwell membrane was observed compared to the medium alone. The migration of livisin peaked at 5 × 10^−10^ M, resulting in approximately 1.4-fold more cells crossing the membrane than untreated keratinocytes (Figure 2a).

To investigate the cytoskeleton changes during keratinocyte migration promoted by livisin, an immunofluorescence assay was performed using phalloidin antibody, which was a fluorescent dye of F-actin filaggrin. The livisin-treated cells exhibited enhanced cell spreading areas, with the pseudopodium area in these cells being twice as large as that of non-treated cells, and the presence of lamellar or needle-like pseudopodia (Figure 2b). These results further confirm livisin has the ability to promote the migration of keratinocytes.

### 2.2. Mechanism of Livisin Promoting Wound Healing

#### 2.2.1. Livisin Activates the CaSR/E-Cadherin Signaling Pathway

**Figure 3:**
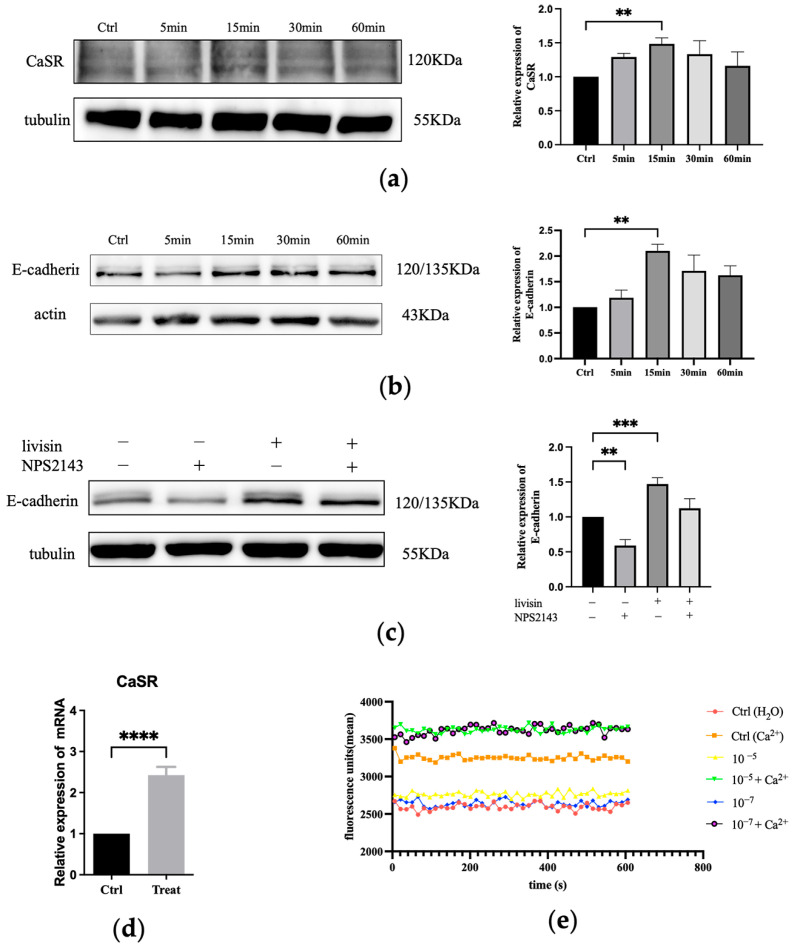
Livisin activates the CasR/E-cadherin signaling pathway. (**a**) Effect of livisin on the protein expression level of CaSR. Keratinocytes were treated with 5 × 10^−10^ M livisin for different durations. The plot besides the blot quantitatively compares the extent of CaSR over time in a grey scale; (**b**) effect of livisin on the protein expression level E-cadherin. Keratinocytes were treated with 5 × 10^−10^ M livisin for different durations. The plot besides the blot quantitatively compares the extent of E-cadherin over time in the grey scale; (**c**) keratinocytes are pretreated with CaSR-specific inhibitor NPS2143 (1 nM) for 12 h, followed by stimulation with 5 × 10^−10^ M livisin. The protein expression of E-cadherin was detected by Western blotting. The plot besides the blot quantitatively compares the extent of E-cadherin in greyscale; (**d**) effect of livisin on the level of CaSR mRNA in HaCaT cells as determined by Q-PCR; (**e**) changes in intracellular Ca^2+^ level of HaCaT cells after treatment with livisin in the absence and presence of 1.2 mM Ca^2+^. In all experiments, the cells were treated with livisin at 5 × 10^−10^ M for 1 h. All graphical data are the means ± SEMs from three determinations. “**”, “***”, and “****” indicate significant differences from the control cells at the *p* < 0.01, *p* < 0.001, and *p* < 0.0001 levels, respectively.

To gain further insight into the mechanism by which livisin promoted cell migration, the impact of livisin on the expression of CaSR and E-cadherin in HaCaT cells was determined. Western blot analysis revealed markedly upregulated levels of CaSR and E-cadherin in HaCaT cells following treatment with livisin (Figure 3a,b). The expression of CaSR reached a peak at 15 min after treatment, with the livisin treatment group exhibiting a 1.5-fold increase compared to the control group. Additionally, q-PCR analysis also demonstrated a significant increase in the level of CaSR mRNA in livisin-treated HaCaT cells, which was 2.4 times higher than that of the untreated group (Figure 3d).

The CaSR-mediated upregulation of E-cadherin was further elucidated by pre-treating HaCaT cells with NPS2143, a specific inhibitor of CaSR, followed by treatment with livisin. In this case, Western blot analysis revealed a significant difference in the expression of E-cadherin between the CaSR-specific inhibitor (NPS2143) group and the livisin treatment group. After blocking CaSR, there was no upregulation of E-cadherin expression, as shown by Western blot analysis (Figure 3c). We next examined the ability of livisin to mobilize intracellular Ca^2+^ using Fluo-4-AM-loaded keratinocytes. The results showed livisin enhanced intracellular Ca^2+^ concentration when co-incubated with Ca^2+^ (Figure 3e). However, it did not cause changes in intracellular Ca^2+^ concentration when exogenous Ca^2+^ was not present.

#### 2.2.2. Livisin Induces Phosphorylation of EGFR and ERK1/2 and Promotes Keratinocyte Migration

**Figure 4:**
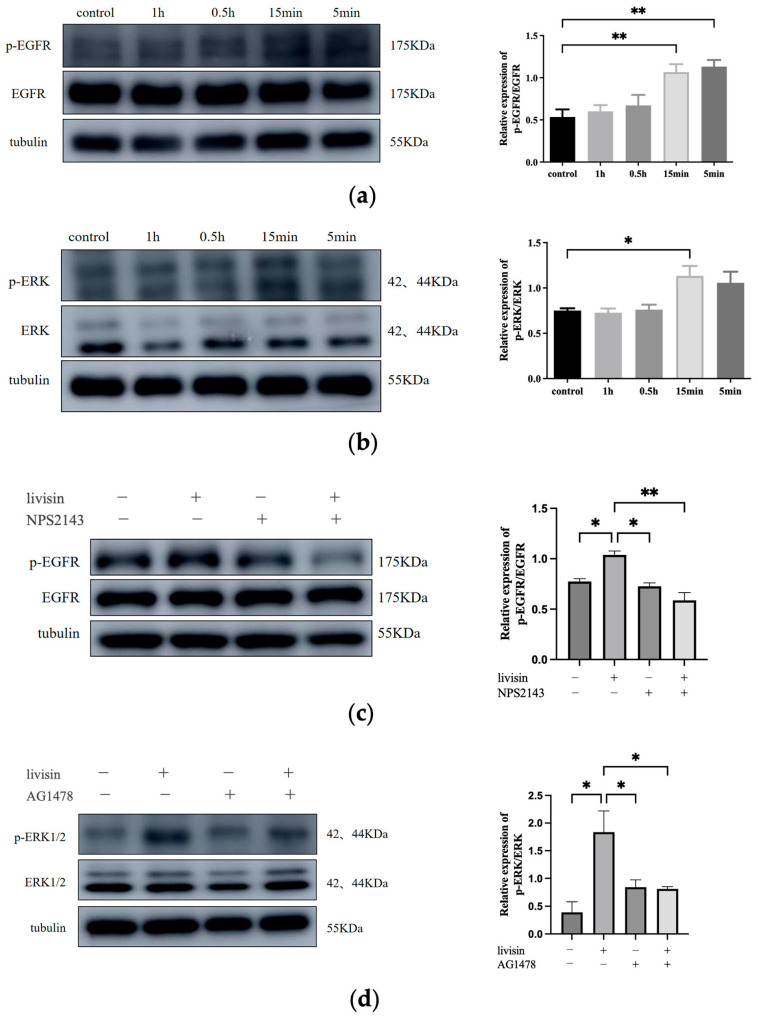
Livisin promotes keratinocyte migration by stimulating the phosphorylation of EGFR and ERK via CaSR. (**a**) Effect of livisin on the phosphorylation of EGFR. The plot besides the blot quantitatively compares the extent of EGFR phosphorylation over time in greyscale; (**b**) effect of livisin on the phosphorylation of ERK. The plot besides the blot quantitatively compares the extent of ERK phosphorylation over time in a grey scale; (**c**) effect of livisin on the phosphorylation of EGFR after blocking CaSR; (**d**) effect of livisin on the phosphorylation of ERK after blocking EGFR. The plot besides the blot quantitatively compares the extent of ERK in a grey scale. All graphical data are the means ± SEMs from three determinations. “*” and “**” indicate significant differences from the control cells at the *p* < 0.05 and *p* < 0.01 levels, respectively.

**Figure 5:**
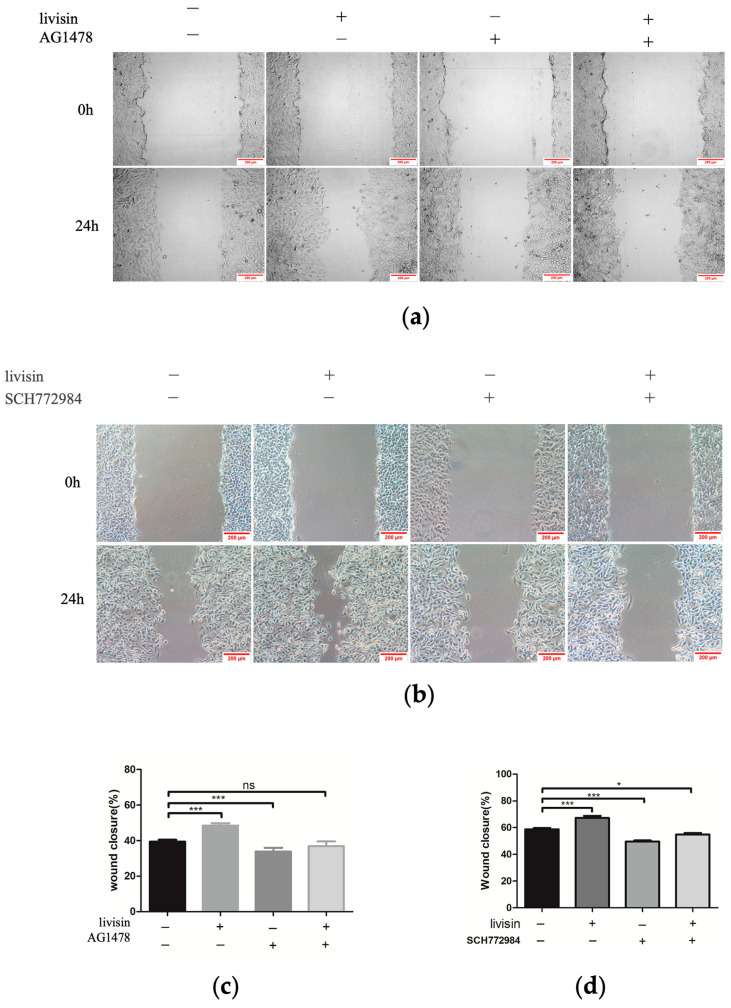
Livisin promotes keratinocyte migration through the EGFR/ERK signaling pathway. (**a**) Effect of livisin on keratinocytes migration after blocking EGFR. The keratinocytes were pretreated with EGFR-specific inhibitor AG1478 (160 nM) for 1 h, then stimulated with 5 × 10^−10^ M livisin. Images were captured immediately after scratching (0 h) and 24 h. Scale bars: 200 μm; (**b**) effect of livisin on keratinocytes migration after blocking ERK1/2. The keratinocytes were pretreated with ERK1/2-specific inhibitor SCH772984 (2 nM), then stimulated with 5 × 10^−10^ M livisin. Images were captured immediately after scratching (0 h) and 24 h. Scale bars: 200 μm; (**c**) three independent results were quantified for (**a**) using ImageJ; (**d**) three independent results were quantified for (**b**) using ImageJ. All graphical data are the means ± SEMs from three determinations. “*” and “***” indicate significant differences from the control cells at the *p* < 0.05 and *p* < 0.001 levels, respectively. “ns” indicates no significant difference.

Previous research has shown a connection between keratinocyte migration and the activation of the EGFR/ERK1/2 signaling pathway [17]. Thus, we aim to investigate if livisin also activates this pathway. When keratinocytes were treated with livisin, we observed increased phosphorylation of EGFR and ERK1/2, reaching peak levels at 15 min. This was 2.4 times higher for EGFR and 1.6 times higher for ERK1/2 compared to the control group (Figure 4a,b). To confirm the role of livisin-mediated EGFR and ERK phosphorylation in keratinocyte migration, CaSR specific inhibitor NPS2143, EGFR specific inhibitor AG1478, and ERK1/2 specific inhibitor SCH772984 were used to perform Western blotting and scratch wound assay. Inhibition of CaSR with NPS2143 abolished the effect of livisin on EGFR phosphorylation, while pretreatment with AG1478 prevented livisin-induced ERK1/2 phosphorylation in HaCaT cells (Figure 4c,d). Scratch assays with HaCaT cells pretreated with AG1478 (EGFR inhibitor) or SCH772984 (ERK1/2 inhibitor) showed a significant reduction in livisin’s ability to promote cell migration compared to cells without inhibitor pretreatment (Figure 5a–d). The results suggested livisin might exert its effect on keratinocyte migration by stimulating the activation of CaSR, which then influenced the EGFR/ERK1/2 signaling pathway. Blocking EGFR/ERK1/2 signaling pathway with specific inhibitors can prevent livisin-induced phosphorylation and subsequently affecting keratinocytes migration.

### 2.3. Livisin Accelerates Wound Healing In Vivo

**Figure 6:**
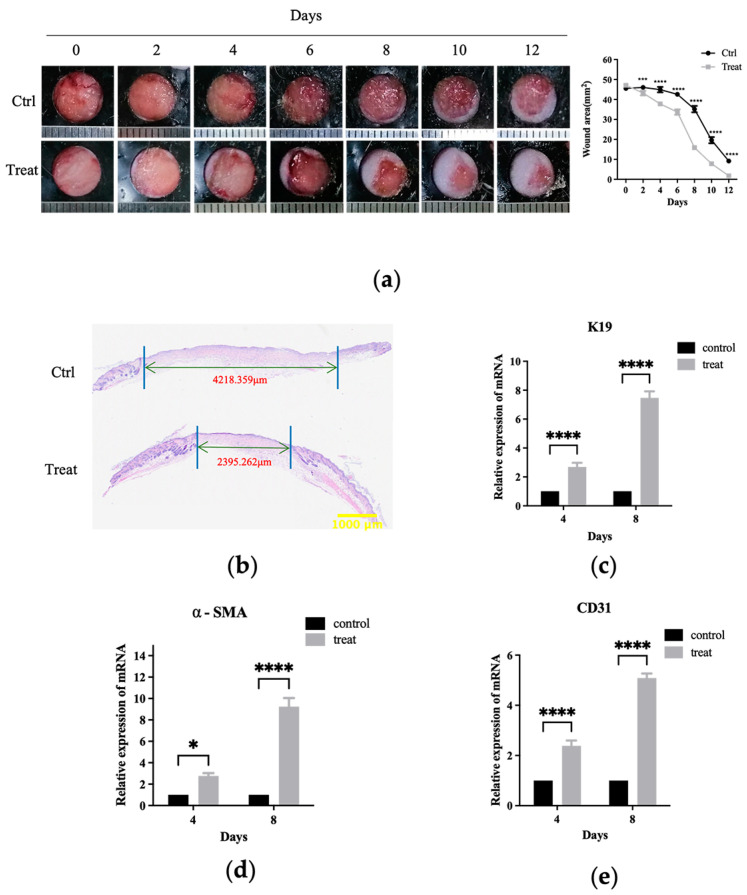
Livisin accelerates the healing of full-thickness wounds in mice. (**a**) The change of wound area after livisin treatment. A total of 50 μL of hydrogel per wound was topically applied to the backs of mice, with or without 500 μM livisin, twice a day. Pictures were taken daily to record the wound-healing process. The plot beside the images compares the change in wound area over time; (**b**) H and E staining of wound sections at 12 days after the operation. The black arrows indicate the edges of the scar. Scale bar: 1000 μm; (**c**) K19 mRNA expression was analyzed at the wound sites on the 4th day and the 8th day post-wound healing; (**d**) α-SMA mRNA expression was analyzed at the wound sites on the 4th day and the 8th day post-wound healing; (**e**) mRNA expression of CD31 at the wound sites on the 4th day and 8th day. All graphical data are the means ± SEMs from three determinations. “*”, “***” and “****” indicate significantly different from the control cells at the *p* < 0.05, *p* < 0.001, and *p* < 0.0001 levels, respectively.

A full-thickness skin wound model was employed to evaluate the recovery process in mice to investigate the influence of livisin on wound healing. To avoid wound shrinking, round rubber washers were used, replicating the human wound-healing process. Local treatment with livisin hydrogel resulted in considerably quicker wound healing compared to the control group (Figure 6a). On the fourth day of healing, a higher rate of wound closure was observed in the livisin-treated group (19.78%) compared to untreated wounds (0.55%). Furthermore, a histological inspection of the wound using H andE staining indicated on the twelfth day, the wound diameter of the control group was about 4.22 mm, and the livisin treatment group was about 2.40 mm. The wound diameter of the control group was about 1.76 times higher than that of the livisin-treated group (Figure 6b). The thickness of the epidermis is a typical measure for assessing wound healing. The findings (Figure S3a) revealed there was a significant difference in the thickness of the wound epidermis between the livisin treatment group and the control group. Livisin increased wound epidermal thickening by about 1.55 times that of the control group on the fourth day, and approximately 1.40 times greater on the eighth day.

The impact of livisin on wound healing was further investigated by analyzing the expression of K19, a keratinocyte marker, in the wound tissue using q-PCR. The results showed significant difference in K19 mRNA expression levels between the livisin treatment group and the control group on the fourth day and eighth day (Figure 6c). The livisin-treated group exhibited a 2.69-fold increase in K19 mRNA expression compared to the control group on the fourth day and a 7.47-fold increase on the eighth day. These findings indicate livisin treatment directly increased K19 mRNA expression, suggesting a potential role in promoting re-epithelialization during wound healing.

Furthermore, the effects of livisin on collagen production, fibroblast transcription, and angiogenesis in the wound tissue were examined. The collagen fibers (blue) and muscle fibers (red) were seen using Masson staining. As shown in Figure S3b, the livisin treatment group had a larger area of blue staining on the fourth day and the eighth day compared to the control group, with a significant difference of 1.35 times and 2.38 times that of the control group, respectively. This indicates livisin treatment increased collagen formation in the wound tissue.

The expression of α-smooth muscle actin (α-SMA), which marked the transition of fibroblasts to myofibroblasts, was also evaluated. The results showed a significant increase in the mRNA expression of α-SMA in the livisin treatment group than that of the control group on the fourth and eighth day of wound healing. Specifically, the treatment group had a 2.77-fold increase in α-SMA mRNA expression compared to the control group on the fourth day and a 9.24-fold higher expression on the eighth day (Figure 6d). These results indicate a substantial upregulation of α-SMA mRNA expression in the livisin-treated group, suggesting a pronounced effect on the transition of fibroblasts to myofibroblasts during wound healing.

CD31, a well-established marker for angiogenesis and tissue regeneration, exhibited a remarkable upregulation in mRNA expression within the livisin treatment group on the fourth and eighth day of wound healing, displaying a 2.39-fold and 5.09-fold increase compared to the control group, respectively (Figure 6e). These findings strongly suggest livisin promotes in vivo wound healing by expediting re-epithelialization, stimulating collagen formation, enhancing fibroblast transcription, and fostering angiogenesis.

## 3. Discussion

The process of skin wound healing is precise and complex and involves inflammation, tissue regeneration, remodeling, and various other processes. Multiple cell types play a role in governing this process, while several kinds of cytokines regulate the wound-healing cascade [18]. Patients must deal with heavy financial and physical burdens due to delayed wound healing and the emergence of chronic wounds [19]. Amphibians, which are known for having exceptional wound-healing abilities, offer potential as an important source for identifying natural compounds that can accelerate wound healing. In this study, we investigated the potential of livisin, a naturally occurring amphibian-derived Bowman–Birk-like trypsin inhibitor (BBLTI) isolated from frog skin secretion, to promote wound healing.

The Bowman–Birk inhibitor (BBI) could be upregulated in response to plant damage or the presence of pathogens, has been recognized for its inherent defensive capabilities in plants [20]. A conserved sequence of CTP1SXPPXC (where P1 is the inhibitory active site and X represents various amino acids) is shared by the peptides in this family, which are primarily obtained from legume seeds and plants [21]. Among these peptides, the disulfide-bridged CWTP1SXPPXPC peptide has been identified as a Bowman–Birk-like trypsin inhibitor (BBLTI) and was discovered in the skin of amphibians [20].

In our previous research [16], we isolated and identified livisin, a novel peptide derived from *Odorrana livida* skin secretions. Livisin is a short peptide containing 17 amino acid residues and two cysteine residues that form disulfide bonds. Research has revealed livisin was an amphibian-derived BBLTI-type peptide with a conserved sequence and serine protease inhibitory activity. Other protease-inhibiting peptides from frog skin have also been studied. For instance, the peptide leucine-arginine (pLR) from the Northern Leopard frog (*Rana pipiens*) exhibits anti-inflammatory activity. It is a histamine-releasing peptide that is non-cell soluble with double the action of melittin, and it can inhibit granulocyte production [22]. Furthermore, the anti-cancer activities of the BBI-type peptide PE-BBI, isolated from the skin of *Pelophylax esculentus*, have been identified [23]. Livisin and other frog skin-derived protease-inhibiting peptides share a common disulfide bridge ring structure. However, research on amphibian-derived BBI-type peptides that stimulate wound healing is limited. We investigated the impact of naturally occurring peptides on wound healing in the context of frogs’ natural habitat. Our findings demonstrated the beneficial effect of livisin helpful on wound healing. Consequently, this study focused on elucidating the function and mechanism of livisin in wound healing, as well as providing unique insights into the application of peptides from this family in wound-healing research.

Both in vivo and in vitro outcomes in the study indicate livisin can enhance angiogenesis at wound sites (Figure 1c and Figure 6e). Interestingly, the angiogenic action of livisin was found to be similar to that of CW49, a host defense peptide from the frog *Odorrana grahami*. CW49 has shown promising therapeutic effects in chronic wound healing in diabetic mice [24]. This suggests livisin may hold therapeutic potential for persistent wounds. Keratinocyte migration and proliferation are critical for wound closure and re-epithelization. Repairing the epidermal barrier promptly can effectively resist the invasion of foreign germs on the wound [18]. Our findings revealed livisin could stimulate keratinocyte migration but not differentiation. Examples of peptides that have been shown to induce cell migration in vitro include hBD-3 [18,25], DRGN-1 [26], Tylotoin [27], TP2-5, and TP2-6 [28], at effective doses ranging from 0.25 to 50 μg/mL. Surprisingly, livisin was found to be effective at a concentration of 1/1000 of these peptides, indicating a potentially greater pharmacological impact. In addition, keratinocytes (Figure 1b), fibroblasts (Figure S1b), and endothelial cells (Figure S2b) were all observed to migrate more readily when exposed to livisin, indicating the broad spectrum of cell migration-promoting properties of this peptide.

The calcium-sensitive receptor (CaSR) is involved in sensing extracellular Ca^2+^ and regulating various physiological activities. Ca^2+^, a critical regulator of keratinocyte differentiation, travels rapidly at wound sites and induces CaSR expression to initiate epithelial healing. Previous studies have indicated E-cadherin acted as a downstream effector of CaSR in the regulation of keratinocyte survival, adhesion, and differentiation [29]. Both proteins are involved in the collective migration of keratinocytes during re-epithelialization. The role of E-cadherin in the activation of EGFR has been established [30,31] and cell migration and differentiation during wound re-epithelialization were carefully controlled by growth factors and the signaling cascades they activated, such as the EGFR/ERK signaling axis [29]. In this study, we investigated the effects of livisin on the CaSR/E-cadherin/EGFR/ERK signaling pathway to elucidate the mechanism through which livisin enhances keratinocyte migration. Our results showed livisin activated the CaSR/E-cadherin/EGFR/ERK signaling pathway (Figure 3 and Figure 4). Surprisingly, the up-regulation of E-cadherin protein expression was not observed after the application of CaSR-specific inhibitors (Figure 3c).

In our investigation we aimed to understand the mechanism of livisin-induced CaSR activation by studying its ability to mobilize intracellular Ca^2+^. Cinacalcet (Sensipar; Amgen Inc., Thousand Oaks, CA, USA) is an approved calcimimetic that acts as an allosteric modulator of CaSR, boosting the action of extracellular calcium by decreasing the receptor activation threshold [32]. However, it requires the presence of extracellular calcium to function [33]. AMG 416 is a synthetic polypeptide calcium mimetic that promotes CaSR activation via a covalent disulfide link between D-cysteine in AMG 416 and CaSR cysteines 482 [34,35]. Its activity is also greatly diminished in the absence of extracellular calcium. In our experiments, cells were incubated with livisin at escalating doses in the presence or absence of 1.2 mM calcium to investigate the relationship between livisin activity and extracellular calcium. The results indicated livisin’s activity was dependent on the presence of extracellular calcium, as there was no discernible change in response values between the control group and the group receiving livisin treatment without calcium (Figure 3e). Contrarily, variations in the presence of calcium were seen, suggesting the presence of extracellular calcium was necessary for livisin to function.

Furthermore, our investigation suggested livisin activated CaSR and the signal was greatly elevated in the presence of calcium, even though there was a relative dearth of current research on natural polypeptide calcifiers. These investigations led to the hypothesis that livisin might act as a calcium-mimetic natural peptide. To explore this further, molecular docking was used to predict the interactions between livisin and CaSR. Previous studies have shown γ-Glutamylpeptide could bind to the VFT domain of CaSR, and the VFT domain of human CaSR protein, which was responsible for ligand binding, ranged from Pro22 to Ile528 [36,37]. The docking results showed the lowest free energy value of the models was −7.3 kcal/mol. Lys9 in livisin formed a hydrogen bond with Tyr310 of CaSR, Arg 4 in livisin formed a hydrogen bond with Ile503 of CaSR, and two more hydrogen bonds were formed between Gly1 in livisin and Ser403 and Asp500 of CaSR (Figure S4). We have preliminarily discovered livisin possessed four hydrogen bonds in the active site of the CaSR VFT domain, which may help to boost CaSR activity. However, further research is needed to confirm this hypothesis and fully understand the mechanism of livisin-induced CaSR activation.

Wound healing relies on the proliferation, migration, and differentiation of relevant cells at the wound site following skin trauma, leading to the generation of new tissues and eventually re-epithelialize [38]. Additionally, in vivo investigations have demonstrated local wound treatment with livisin accelerated skin wound healing in a full-layer skin wound model in mice (Figure 6), dramatically enhanced collagen formation, and stimulated the growth of granulation tissue (Figure S3). Moreover, in vivo experiments showed livisin significantly upregulated the expression of smooth muscle cell differentiation marker α-SMA (Figure 6d) and the angiogenesis marker CD31 (Figure 6e), suggesting livisin had the potential to expedite the transformation of fibroblasts into myofibroblasts and promote angiogenesis, ultimately facilitating wound closure.

## 4. Conclusions

In this study, we conducted a comprehensive investigation into the wound-healing properties of livisin at the cellular, molecular, and individual levels. Our findings demonstrate livisin significantly enhances the migratory capacity of keratinocytes and accelerates wound recovery in mice. We propose the activation of the CaSR/E-cadherin/EGFR/ERK signaling pathway may underlies these effects, with livisin acting as a natural peptide calcimimetic that activates CaSR. Furthermore, livisin promotes collagen deposition, fibroblast-to-myofibroblast differentiation, angiogenesis, and re-epithelialization, collectively expediting the process of wound healing. In conclusion, our study provides compelling experimental evidence supporting the use of livisin in the treatment of skin wound, highlighting its potential as an effective therapeutic option.

## 5. Materials and Methods

Solid-phase peptide synthesis. Livisin, which has the sequence GFLRGCWTKSFPPKPCL, was synthesized by solid-phase peptide synthesis with a purity of over 95%. The peptide was synthesized and purified by GenScript Biotech Corporation. The structure and properties of the synthesized peptide were the same as those found in the natural peptide [16].

Cell culture. Human immortalized keratinocytes (HaCaT cell line) and mouse fibroblasts (L929) were purchased from the Institute of Biochemistry and Cell Biology, Chinese Academy of Sciences, and cultured in DMEM (Gibco, Carlsbad, CA, USA) supplemented with 10% (*v*/*v*) fetal bovine serum (TransGen Biotech, Beijing, China) and antibiotics (100 U/mL penicillin and 100 μg/mL streptomycin (Gibco, Carlsbad, CA, USA). Human microvascular endothelial cell HMEC-1, purchased from the Institute of Biochemistry and Cell Biology, Chinese Academy of Sciences, was cultured in RPMI 1640 (Gibco, Carlsbad, CA, USA) supplemented with fetal bovine serum and antibiotics (as mentioned above). All cells were cultured at 37 °C in a 5% CO_2_ incubator.

Proliferation assay. The effect of livisin on cell proliferation was determined by the MTT assay. Briefly, HaCaT, L929, and HMEC-1 cells were seeded in a 96-well plate at a density of 8000 cells/well (six replicates per group) in a medium supplemented without and with different concentrations of livisin (1 × 10^−5^–1 × 10^−11^ M). The plate was incubated at 37 °C for 24 h. Next, MTT (Sangon Biotec, Shanghai, China) reagent was added to the cells (20 μL/well), and the plate was incubated for another 4 h. After that, DMSO (Sangon Biotec, Shanghai, China) was added (100 μL/well) and the absorbance was measured at 490 nm using a microplate reader (Bio-Rad 680, Bio-Rad, Hercules, CA, USA).

Migration assay. The effect of livisin on cell migration was investigated using the scratch wound assay and the transwell assay. For the scratch wound assay, HaCaT cells were plated in a 6-well plate at a cell density of 1 × 10^6^ cells/well (three replicates per group) and incubated until confluent. The monolayer was scratched using a pipette tip and washed with a serum-free medium to remove the detached cells, followed by incubation in a complete medium without or with livisin (5 × 10^−11^, 5 × 10^−10^ M) and an EGFR inhibitor AG1478 (Selleck, Houston, TX, USA) or ERK inhibitor SCH772984 (Selleck, Houston, TX, USA). The cells were photographed (Leica DMI6000B, Vizsla, Germany) at 0 h and 24 h post-wounding. The closure area of the wound was calculated as follows: migration area (%) = (A0 − An)/A0 × 100, where A0 is the area of the initial wound area and An is the remaining area of the wound at the metering point. In the case of the transwell assay, HaCaT cells were suspended in serum-free DMEM medium containing no livisin or livisin (5 × 10^−9^, 5 × 10^−10^ M) and seeded into the upper chamber of a transwell in a 24-well transwell plate (6.5 mm diameter polycarbonate filter with 8.0 μm pore size, Corning, NY, USA) at a cell density of 4 × 10^5^ cells/well (three replicates per group). Then the lower chamber was filled with a complete medium supplemented with 10% FBS. After 48 h of incubation, the cells attached to the upper surface of the filter membranes were carefully removed, and migrated cells at the lower surface were stained with 0.5% crystal violet for 10 min and observed with a microscope (Leica DMI6000B, Vizsla, Germany). The crystal violet was dissolved with 33% glacial acetic acid, and the absorbance was observed at 590 nm using a microplate reader (Bio-Rad 680, Bio-Rad, Hercules, CA, USA).

Tube formation assay. The tube-forming assay was performed according to a previously described method [39]. Matrigel matrix was added to each well of a 24-well plate (289 μL/well) and incubated at 37 °C for 30 min. Meanwhile, HMEC-1 cells were detached by treatment with trypsin, washed, and then resuspended in a serum-free medium. The cell suspension was seeded into a 24-well plate containing the Matrigel matrix at a cell density of 4 × 10^5^ cells/well. The cells were incubated with 10% FBS (as a positive control), vehicle (as a negative control), or 1 × 10^−5^ M livisin for 18 h at 37 °C in a 5% CO_2_ incubator. Tubes were photographed using a microscope (Leica DMI6000B, Vizsla, Germany), and the number of nodes was analyzed using Image J software 1.53 (National Institutes of Health, Bethesda, MD, USA).

Immunofluorescence staining. Cells on coverslips were fixed with 4% paraformaldehyde for 15 min, washed with PBS three times, permeabilized in 0.5% Triton X-100 (Sangon Biotech, Shanghai, China) in PBS for 30 min, and then incubated with blocking solution (5% goat serum in 1 × PBS) for 1.5 h at room temperature. Phalloidin-Alexa Fluor 555 (Beyotime Biotechnology, Shanghai, China) was used to stain the cytoskeleton. The slides were mounted with Vectashield/DAPI stain (Vector Laboratories H-1200) and stored at 4 °C in the dark. The cells were imaged using a Nikon microscope.

Western blotting. The cells were lysed in RIPA (Beyotime Biotechnology, Shanghai, China) containing Protease Inhibitors Cocktail and Phosphatase Inhibitors Cocktail (TransGen Biotech, Beijing, China). The cell extract was then resolved in 10% SDS-PAGE gels followed by Western blot analysis as described previously [25]. Preincubation with CaSR inhibitor NPS2143 (Selleck, Houston, TX, USA) for 12 h, EGFR inhibitor AG1478 (Selleck, Houston, TX, USA) for 2 h before detecting E-cadherin, ERK, p-ERK, EGFR, and p-EGFR expression. Western blot was carried out with a rabbit primary antibody against CaSR (Affinity Biosciences, Cincinnati, OH, USA), E-cadherin (Abcam, Cambridge, UK), ERK (Abcam, Cambridge, UK), p-ERK (Abcam, Cambridge, UK), EGFR (Abcam, Cambridge, UK), or p-EGFR (Abcam, Cambridge, UK) and anti-rabbit HRP-conjugated antibody as the secondary antibody. The blot was developed with ECL reagent (Proteintech, Chicago, IL, USA) and visualized by Amersham™ Imager 600 gel imaging system (Cytiva, Boston, USA). 

The intracellular concentration of the Ca^2+^ determination assay. Changes in intracellular Ca^2+^ concentration were detected with the calcium indicator fluo-4 AM (Beyotime Biotechnology, Shanghai, China). First, HaCaT cells were grown in a petri dish in a complete medium until about 80% confluent. The medium was then discarded, and the cells were washed three times with PBS. Next, the cells were resuspended in a 15-mL centrifuge tube and washed three times with PBS, and 1 mL of Fluo-4 AM (2 μM) was then added to the cells, followed by incubation at 37 °C for 30 min in the dark. After that, the cells were washed three times with PBS and resuspended in PBS. The cell suspension was dispensed into a black 96-well plate using 50 mL per well, followed by the addition of 50 mL livisin solution with or without CaCl_2_ (1.2 mM). The final concentration of livisin in the cell sample ranged from 10^−5^ to 10^−7^ M. Finally, the fluorescence of the plate was measured continuously for 10 min at every 15-sec interval using excitation and emission wavelengths of 488 nm and 516 nm, respectively.

Quantitative real-time RT-PCR analysis. TRIzol (Invitrogen, Carlsbad, CA, USA) was used to extract total RNA from the cultured HaCaT cells and tissues. The extracted total RNA was used to synthesize the cDNA using an EasyScript^®^ All-in-One First-Strand cDNA Synthesis SuperMix for qPCR reagent kit (TransGen Biotech, Beijing, China) according to the manufacturer’s instructions. The gene names and corresponding forward and reverse primer pairs were used: hCaSR: 5′-ACCAGCGAGCCCAAAAGAAG-3′ and 5′-GACTCCGGCCTTGATTTGAGA-3′; hGAPDH: 5′-TGCACCACCAACTGCTTAGC-3′ and 5′-GGCATGGACTGTGGTCATGAG-3′; mCaSR: 5′-GCATCAGGTATAACTTCCGTGG-3′ and 5′-CGGTGTTACAGGTGTCGAATATC-3′; mα-SMA: 5′-GGCACCACTGAACCCTAAGG-3′ and 5′-ACAATACCAGTTGTACGTCCAGA-3′; mCD31: 5′-ACGCTGGTGCTCTATGCAAG-3′ and 5′-TCAGTTGCTGCCCATTCATCA-3′; mK19: 5′-GTTCAGTACGCATTGGGTCAG-3′ and 5′-GAGGACGAGGTCACGAAGC-3′; mGAPDH: 5′-AGGTCGGTGTGAACGGATTTG-3′ and 5′-GGGGTCGTTGATGGCAACA-3′. The fold difference in gene expression was normalized to the housekeeping gene GAPDH.

Mouse wound-healing model. Hydrogels containing no livisin or 500 μM livisin were prepared, and the composition of the hydrogel was shown in Table S1. Six-eight-week-old male ICR mice with weights ranging from 30–35 g were provided by the Animal Laboratory Center of Wenzhou Medical University. The mice were housed in aseptic rooms at the Animal Facility at Wenzhou University under a 12-h light/dark cycle at 22 °C. Each animal was kept in a cage laid with a layer of sawdust, which was changed daily. After a week of acclimatization, the mice were weighed, and the body weight was recorded. After the mice were anesthetized with 2.5% isoflurane, the back skin hair was shaved, and two sterile silicone gaskets with a diameter of 1.6 cm and a thickness of 0.05 cm were sutured on both sides of the back skin of the mice under sterile conditions to avoid and prevent wound contraction. Two full-thickness wounds with a diameter of 8 mm were made along the inner ring of the gasket with sterilized surgical scissors, and the wounds were then wrapped with sterile gauze. The mice were randomly divided into two groups. The first group was treated with hydrogel only. In this group, the wound in the animal was topically applied with 50 μL hydrogel twice a day for 12 days. The second group was treated with the hydrogel containing 500 μM livisin. In this group, the wound was topically applied with 50 μL of livisin-containing hydrogel twice a day for 12 days. Each wound was digitally photographed, and the wound area was calculated using the ImageJ software. The mice were eventually sacrificed, and the wound skin samples were collected for further experiments. All animals were handled according to the international animal care and handling guidelines provided by the International Conference on Harmonization (ICH) and the Organisation for Economic Cooperation and Development (OECD).

Tissue preparation and histological analysis. Wound tissues were fixed in 4% paraformaldehyde for 24 h. After dehydration, the tissue samples were embedded in paraffin wax and cut into slices of 5 μm thickness. The slices were subjected to H and E and Masson staining, followed by microscopic examination to observe the wound width, new epidermal thickness, and collagen production.

Molecular simulation and docking. The structure of CaSR (PDB ID: 5FBK) was obtained through the RCSB website, and Pymol was further used to remove ligands and water molecules from the protein. The 3D model of livisin was predicted with the aid of the I-TASSER web server. The overall quality of the model was quantified using the z-score of ProSA. The missing polar hydrogens were added to the peptide model using Autodock Tools. After energy minimization, livisin was docked with CaSR using the high-resolution peptide-protein docking tool FlexPepDock implemented in the Rosetta framework. NCI analysis was performed using the Multiwfn software to study the relationship between the residues around the active site.

Statistical analysis. All experiments were performed at least three times. Statistical analysis was performed using one-way or two-way analysis of variance (ANOVA) with a multiple comparisons test or Student’s *t*-test using GraphPad Prism version 9.0 (GraphPad Software Inc., San Diego, CA, USA). Statistical significance was considered at the *p* < 0.05 level.

## Data Availability

Data are contained within the article and Supplementary Materials.

## Supplementary Materials

The following supporting information can be downloaded at: https://www.mdpi.com/article/10.3390/toxins16010021/s1. Figure S1: Livisin promotes fibroblast L929 migration but does not affect its proliferation. Figure S2: Livisin promotes human microvascular endothelial cells HMEC-1 migration but does not affect its proliferation. Figure S3: Livisin can accelerate the formation of new wound epithelium and collagen deposition. Figuer S4: The molecular docking of livisin (gray) with CaSR (cyans, PDB code:5FBK). Table S1: Hydrogel matrix formulation.
