# Supplementary material for: Revitalizing Skin Repair: Unveiling the Healing Power of Livisin, a Natural Peptide Calcium Mimetic
Source: Toxins (Basel). 2023 Dec 31;16(1):21. doi: 10.3390/toxins16010021 (PMC10819626; doi:10.3390/toxins16010021)
Supplement: Supplementary file 1 [file toxins-16-00021-s001.zip › toxins-2720602-supplementary.pdf]

Supplementary Materials

Figure S1

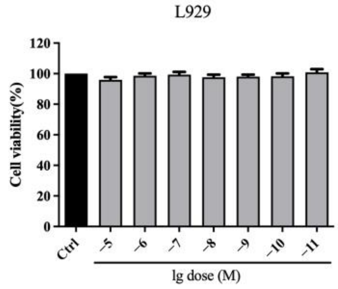

(**a**)

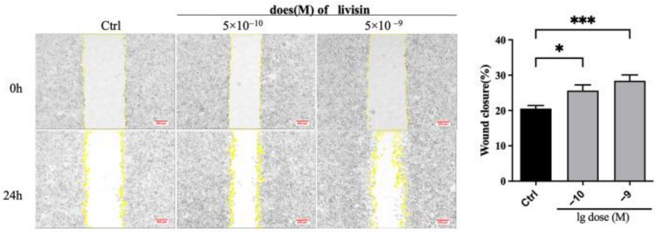

(**b**)

Figure S1. Livisin promotes fibroblast L929 migration but does not affect its proliferation.

Figure S2

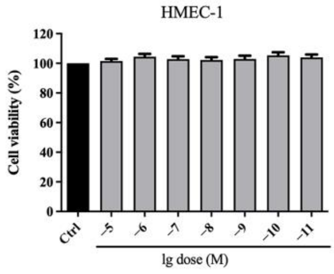

(**a**)

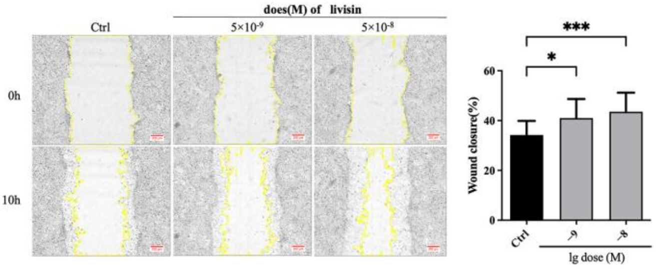

(**b**)

Figure S2. Livisin promotes human microvascular endothelial cells HMEC-1 migration but does not affect its proliferation.

Figure S3

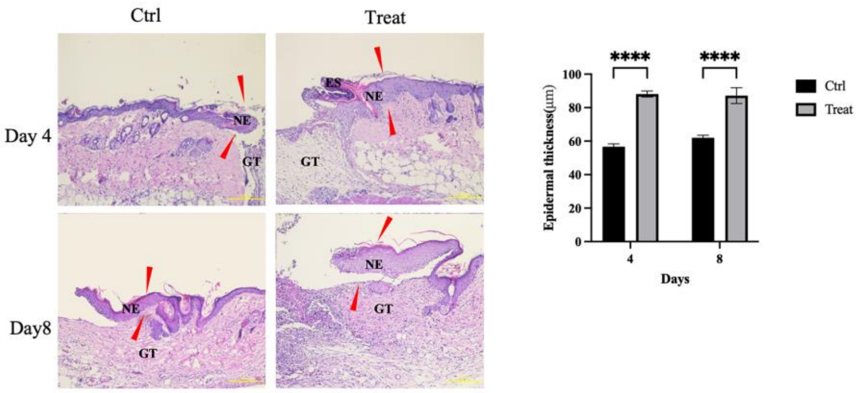

(a)

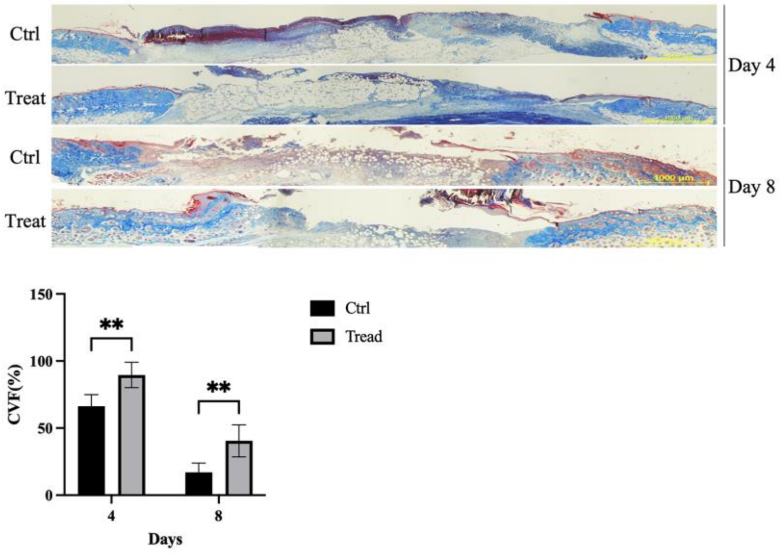

(b)

Figure S3. Livisin can accelerate the formation of new wound epithelium and collagen deposition.

Figure S4

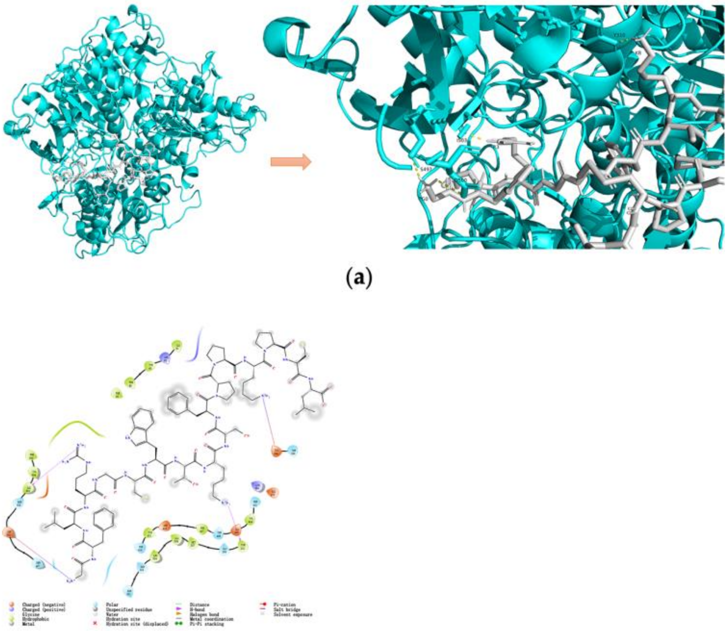

(b)

Figure S4. The molecular docking of livisin (gray) with CaSR (cyans, PDB code:5FBK).

Table S1 Hydrogel Matrix Formulation

Name	Dose
Glycerin	5mL
ddH2O	40mL
Kapom940	0.25g
Triethanolamine	0.28g
